# Electron-transfer-initiated benzoin- and Stetter-like reactions in packed-bed reactors for process intensification

**DOI:** 10.3762/bjoc.12.268

**Published:** 2016-12-13

**Authors:** Anna Zaghi, Daniele Ragno, Graziano Di Carmine, Carmela De Risi, Olga Bortolini, Pier Paolo Giovannini, Giancarlo Fantin, Alessandro Massi

**Affiliations:** 1 Dipartimento di Scienze Chimiche e Farmaceutiche, Università di Ferrara, Via Fossato di Mortara 17, I-44121 Ferrara, Italy

**Keywords:** C–C coupling, continuos-flow, diketone, electron-transfer, umpolung

## Abstract

A convenient heterogeneous continuous-flow procedure for the polarity reversal of aromatic α-diketones is presented. Propaedeutic batch experiments have been initially performed to select the optimal supported base capable to initiate the two electron-transfer process from the carbamoyl anion of the *N*,*N*-dimethylformamide (DMF) solvent to the α-diketone and generate the corresponding enediolate active species. After having identified the 2-*tert*-butylimino-2-diethylamino-1,3-dimethylperhydro-1,3,2-diazaphosphorine on polystyrene (PS-BEMP) as the suitable base, packed-bed microreactors (pressure-resistant stainless-steel columns) have been fabricated and operated to accomplish the chemoselective synthesis of aroylated α-hydroxy ketones and 2-benzoyl-1,4-diones (benzoin- and Stetter-like products, respectively) with a good level of efficiency and with a long-term stability of the packing material (up to five days).

## Introduction

**Figure 1:**
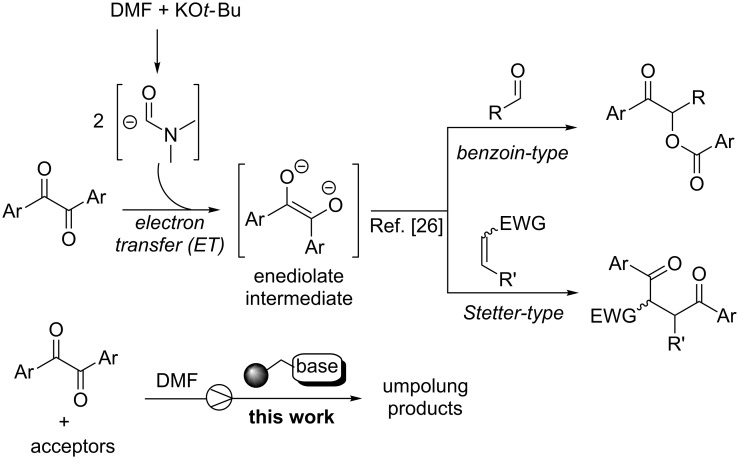
Electron-transfer initiated activation of α-diketones (background) and present study.

The polarity reversal (umpolung) of carbonyl compounds by N-heterocyclic carbene (NHC) or cyanide catalysis represents a straightforward strategy for the synthesis of valuable molecules such as, among the many examples, α-hydroxy ketones (benzoin reaction) and 1,4-diketones (Stetter reaction) [1–4]. The synthetic utility of the umpolung methodology has therefore spurred intensive research on process intensification through the heterogeneization of NHC catalysts [5–9] for facilitating the post-reaction phase and improving NHCs’ stability towards air and moisture [10–11]. Quite surprisingly, however, implementation of continuos-flow techniques with micro- and meso-reactors is rare in this field [12–17]. Indeed, microreactor technology is today a powerful tool for the fine chemical and pharmaceutical industries facilitating the automation of the production processes with reduced costs and improved safety and sustainability [18–21]. Very recently, Monbaliu and co-workers described a convenient continuous-flow setup for the generation of common free NHCs under homogeneous conditions and their subsequent utilization in transesterification and amidation processes by the reaction telescoping approach [12]. Similarly, the group of Brown reported on the oxidative esterification and amidation of aldehydes in undivided microfluidic electrolysis cells mediated by homogeneous NHCs [13–14]. On the other hand, heterogeneous catalysis in microstructured flow reactors represents a robust synthetic platform, with benefits over the corresponding batch processes such as catalyst stability, lower degradation of supports, and ease of scale-up with minimal changes to the reaction setup [22–24]. An integrated flow system for the synthesis of biodiesel employing an uninterrupted sequence of two fixed-bed reactors packed with a supported acid for esterification of free fatty acids and with an immobilized imidazolidene catalyst for transesterification has been recently described by Lupton and co-workers [15]. Our group also contributed to this area of research fabricating polystyrene monolithic columns functionalized with thiazolium salt pre-catalysts to perform umpolung racemic processes (benzoin, acyloin, and Stetter reactions) with a good level of efficiency [16]. The asymmetric version of acyloin-type reactions was also investigated in our laboratory operating packed-bed bioreactors functionalized with a suitable thiamine diphosphate (ThDP)-dependent enzyme supported on mesoporous silica [17]. Overall, the so far reported umpolung flow processes [12–17] required quite sophisticated procedures, eventually complicated by the separation of homogeneous azolium salt pre-catalysts [25]. In this contribution, we describe a convenient and straightforward continuos-flow protocol for the effective production of benzoin and Stetter-like products that relies on the use of a readily and commercially available supported base as packing material of fixed-bed microreactors. The present study originated from our recent findings on a novel strategy for the umpolung of aromatic α-diketone donors [26] and their peculiar reactivity with aromatic aldehydes or α,β-unsaturated acceptors [27–29]. Indeed, activation of aromatic α-diketones may occur through a double electron-transfer (ET) process triggered by the carbamoyl anion derived from *N*,*N*-dimethylformamide (DMF) solvent with catalytic base, which generates an enediolate anion as key reactive species of umpolung catalysis (Figure 1). Significantly, the current investigation on the heterogeneous continuous-flow version of the α-diketone activation process resulted in the fabrication of fixed-bed reactors with elevated stability, allowing their operation for about five days with maintenance of productivity. Moreover, the disclosed flow procedure constituted an equally effective (complete chemoselectivity) and environmentally benign alternative to the analogous batch process towards benzoin- and Stetter-type products mediated by toxic cyanide anions [29–30].

## Results and Discussion

The possibility of transposing the ET-mediated activation process of aromatic α-diketones (benzils) from a homogeneous batch protocol to a heterogeneous flow procedure was initially investigated by testing the efficacy of the commercially available supported bases **4**–**8** under batch conditions; the benzoin-type reaction of benzil **1a** with 2-chlorobenzaldehyde **2a** furnishing the benzoylated benzoin **3aa** (double aroylation product) was selected as the benchmark (Table 1). Quite surprisingly, the polystyrene-supported 1,8-diazabicyclo [5.4.0]undec-7-ene **4** (PS-DBU) was completely inefficient (DMF, 35 °C, Ar atmosphere) in both catalytic and equimolar amounts despite the detected activity of its homogeneous counterpart [26] (Table 1, entries 1 and 2). Gratifyingly, the highly basic, non-nucleophilic polymer-supported BEMP **5** (PS-BEMP: 2-*tert*-butylimino-2-diethylamino-1,3-dimethyl-perhydro-1,3,2-diazaphosphorine on polystyrene) afforded the target adduct **3aa** in almost quantitative yield (95%) when used in equimolar amounts under an argon atmosphere (Table 1, entry 3). Actually, we previously established the importance of operating under deaerated conditions with homogeneous bases to avoid a marked decrease of the reaction rate (vide infra). By contrast, as demonstrated by the experiment of Table 1, entry 4, the **1a/2a** coupling promoted by PS-BEMP **5** was found to be insensitive to the presence of air, thus further improving the practicality of the heterogeneous procedure for the umpolung of benzils. While the utilization of catalytic PS-BEMP **5** (25 mol %) at 35 °C slightly diminished the reaction yield (78%, Table 1, entry 5), the increase of temperature to 50 °C restored the reaction efficiency (91% yield, entry 6). A lower amount of **5** (10 mol %) produced an unsatisfactory yield of **3aa** (28%, Table 1, entry 7), whereas the weaker bases diethylamine resin **6**, Ambersep 900 OH **7**, and the polymer-bound tetraalkylammonium carbonate **8** were completely inefficient (Table 1, entries 8–10). Finally, the conversion efficiency was maintained almost unaltered for recycled PS-BEMP **5** after five runs (Table 1, entry 11). The success of the recycle experiment paved the way for the application of **5** in continuous-flow processes with long-term stability.

**Table 1:** Optimization of the cross-benzoin-type reaction of benzil **1a** with 2-chlorobenzaldehyde **2a** promoted by the supported bases **4**–**8** under batch conditions.^a^

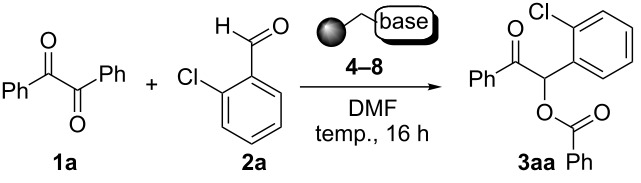			

Entry	Base [mol %]	Temp. [°C]	Yield [%]^b^

1^c^	**4** (25)	35	<5
2^c^	**4** (100)	35	<5
3^c^	**5** (100)	35	95
4	**5** (100)	35	92
5	**5** (25)	35	78
6	**5** (25)	50	91
7	**5** (10)	50	28
8	**6** (100)	50	<5
9	**7** (100)	50	<5
10	**8** (100)	50	<5
11^d^	**5** (25)	50	89

^a^Reaction Conditions: benzil **1a** (0.50 mmol), 2-chlorobenzaldehyde **2a** (0.60 mmol), DMF (1.0 mL; water content 0.23% w/w), and the stated amount of base.
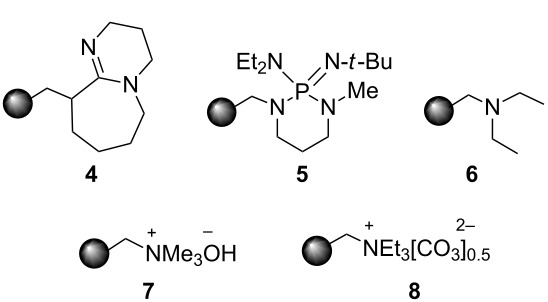
^b^Isolated yield. ^c^Reaction conducted under Ar. ^d^5th recycle.

Next, the heterogeneous procedure for the activation of aromatic α-diketones was applied to the model Stetter-like reaction of benzil **1a** with chalcone **9a** serving as activated α,β-unsaturated acceptor (Table 2). The optimal conditions disclosed for the benzoin-like reaction (25 mol % **5**, 50 °C) were not applicable to the **1a/9a** coupling (Table 2, entry 1). Also, the use of equimolar **5** gave the target 1,4-dione **10aa** in poor yield (26%, Table 2, entry 2) after filtration of **5** and its resuspension in a 10:1 CH_2_Cl_2_–AcOH mixture (30 min, rt). This work-up procedure was made necessary because of the sequestering by the basic resin **5** of compounds of type **10** displaying acidic protons at the α-position of carbonyl groups. A higher product yield (45%) was obtained at 70 °C (Table 2, entry 3), while a further increase of temperature (100 °C) and the use of microwave irradiation at 120 °C (1 h) were not beneficial for the reaction outcome (Table 2, entries 4 and 5). The model Stetter-like reaction was finally optimized by varying the **1a/9a** ratio (Table 2, entries 6 and 7) and the best yield of **10aa** (68%) was achieved at 70 °C with an excess of benzil (**1a**, 2 equiv; Table 2, entry 6).

**Table 2:** Optimization of the Stetter-type reaction of benzil (**1a**) with chalcone **9a** promoted by PS-BEMP **5** under batch conditions.^a^

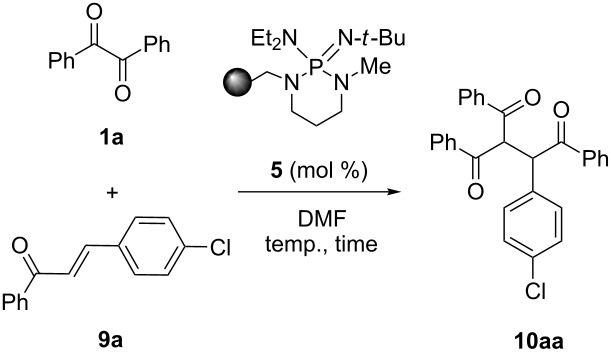				

Entry	**5** [mol %]	Temp. [°C]	Time [h]	Yield [%]^b^

1	25	50	16	<5
2	100	50	16	26
3	100	70	8	45
4	100	100	8	24
5^c^	100	120	1	31
6^d^	100	70	8	68
7^e^	100	70	8	41

^a^Reaction conditions: benzil (**1a**, 0.50 mmol), chalcone (**9a**, 0.50 mmol), DMF (1.0 mL; water 0.23% w/w), and the stated amount of **5**. ^b^Isolated yield. ^c^Reaction warmed by microwave irradiation (Biotage Initiator; temperature was measured externally by an IR sensor). ^d^Reaction performed with 1.00 mmol of **1a**. ^e^Reaction performed with 1.00 mmol of **9a**.

**Scheme 1:**
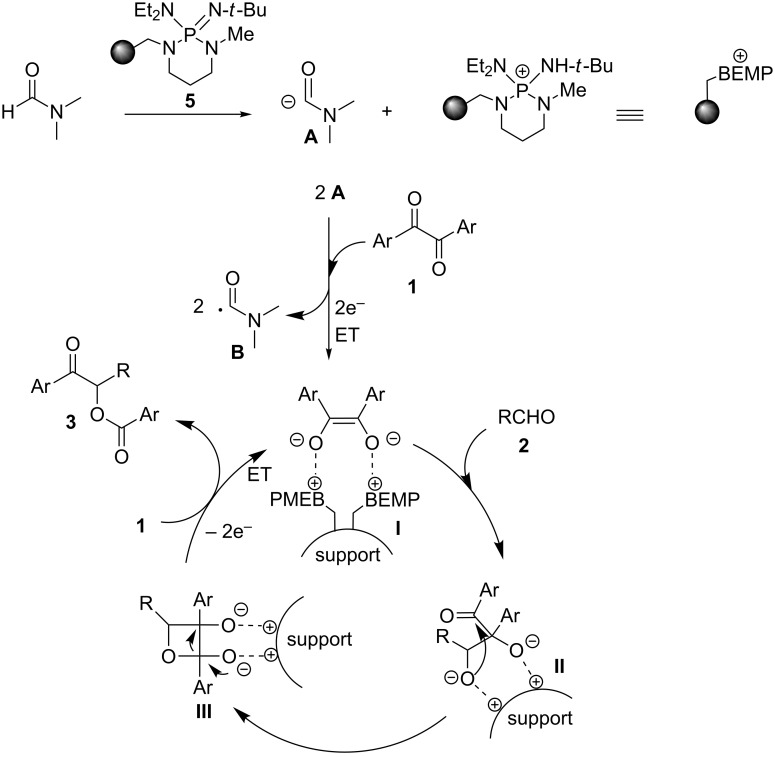
Proposed dianionic pathway for the cross-benzoin-like reaction of benzils **1** with aldehydes **2** under heterogeneous conditions.

On the basis of our previous mechanistic investigation in solution phase [26], the above results may be interpreted as follows. The carbamoyl anion **A**, which is generated by deprotonation of DMF solvent with PS-BEMP **5**, is responsible for two sequential ET to the α-diketone **1** leading to the carbamoyl radical **B** (non-productive pathway) [26] and the key enediolate intermediate **I** bound to the polymer as ion pair (Scheme 1). In the case of benzoin-like reactions, the supported species **I** intercepts the aldehyde acceptor **2** to form the cyclic intermediate **III** through the first adduct **II**. Then, the final two ET from **III** to the α-diketone **1** affords the product **3** regenerating the dianion **I** ready for a chain process. It is important to underline the beneficial effect on the reaction outcome and practicability of the polymer support, which stabilizes the enediolate functionality through ionic interactions, thus preventing the fast oxidation by oxygen of **I** to the α-diketone **1** and the consequent slowing down of the reaction as observed under homogeneous conditions. [26]

**Scheme 2:**
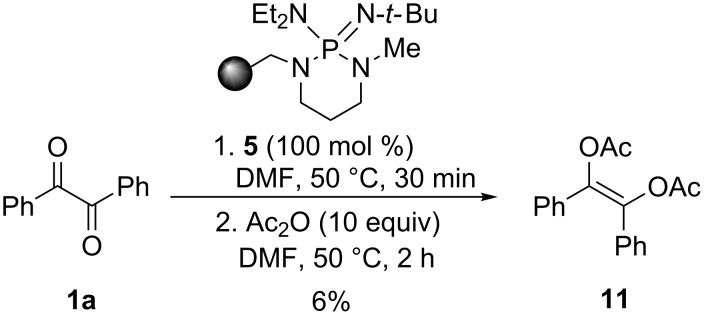
Trapping experiment.

In analogy with the study under homogeneous conditions, a trapping experiment was also performed to confirm the crucial role in the catalytic cycle of the enediolate intermediate **I**. Accordingly, the suspension of benzil (**1a**) and equimolar PS-BEMP **5** in DMF was treated at 50 °C with an excess (10 equiv) of acetic anhydride recovering the expected *O*,*O*’-diacetyl-1,2-diphenylethen-1,2 diol (**11**) in 6% isolated yield (Scheme 2).

At this stage of our investigation, PS-BEMP **5** was tested as the packing material of fixed-bed reactors with potential long-term stability. A micro-HPLC with minimized extra-column volumes was used as the pumping system. The fixed-bed microreactor **R5** was then fabricated by packing a stainless steel column (10 cm length, 4.6 mm internal diameter) with PS-BEMP **5**. Pycnometer measurements provided the hold-up volume *V*_o_ and the total porosity ε_tot_ of **R5** [31], whereas the loaded amount of **5** was determined by weighing the filled and empty column. The main features of **R5** including the residence time and the observed backpressure are summarized in Table 3.

**Table 3:** Main features of microreactor **R5**.^a^

Packed **5** [g]	**5** Loading [mmol/g]^b^	*V*_0_ [mL]^c^	Total porosity^d^	Time [min]^e^	Pressure [bar]^f^

0.99	2.20	1.38	0.83	138	4

^a^Geometric volume (*V*_G_) of the stainless-steel column: 1.66 mL. ^b^Value given by the supplier. ^c^Determined by pycnometry (see the Experimental section). ^d^Total porosity ε_tot_ = *V*_0_*/V*_G_. ^e^Residence time calculated at 10 μL min^−1^. ^f^Backpressure measured at 10 μL min^−1^ (DMF, 50 °C).

**Figure 2:**
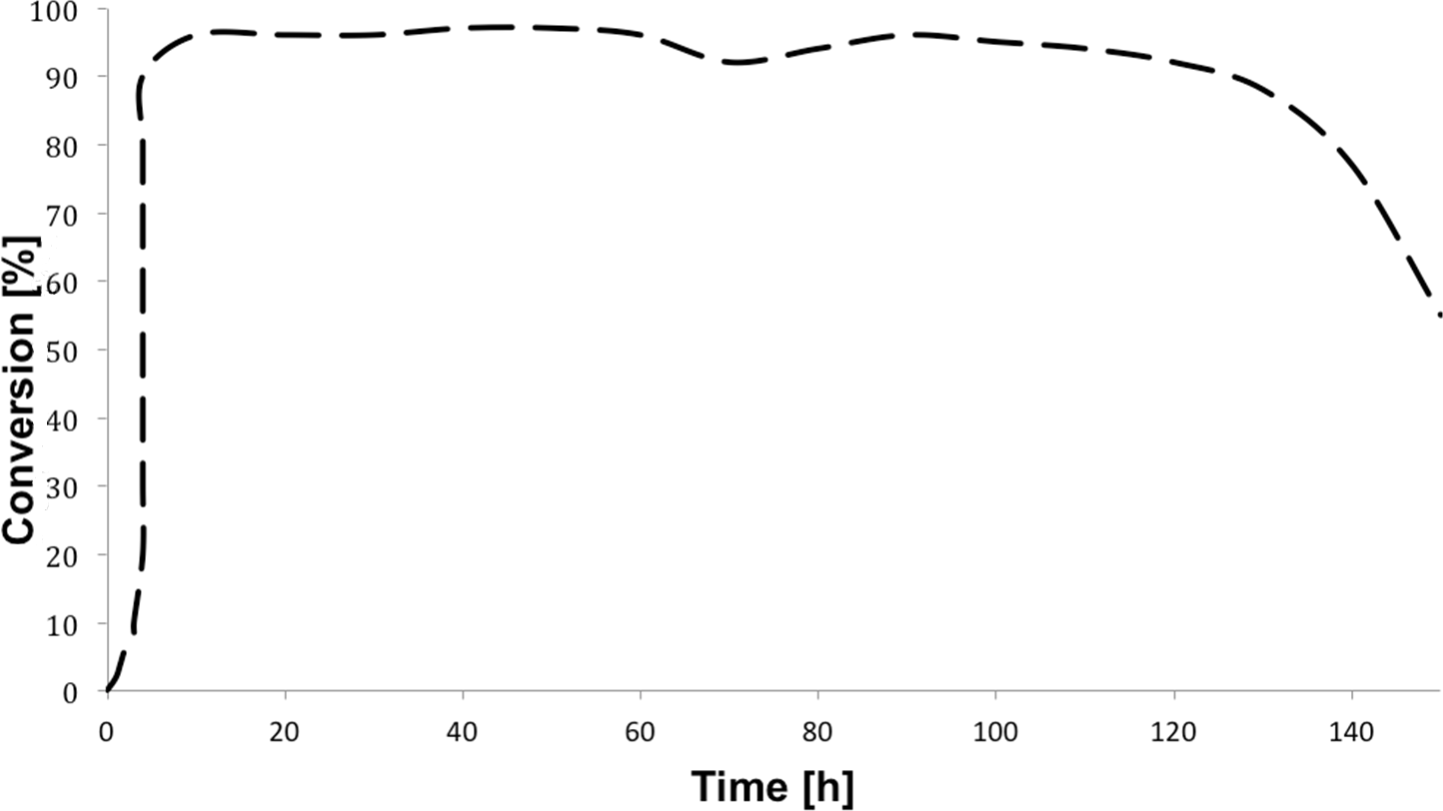
Conversion of the **1a/2a** coupling in microreactor **R5** operated for 150 h at 50 °C.

Continuous-flow experiments were performed by first considering the benzoin-like reaction of benzil (**1a**) with 2-chlorobenzaldehyde (**2a**) (Table 4). Different flow rates and substrate concentrations were initially evaluated to optimize the conversion efficiency and productivity (*P*) of the process. Hence, portions of the outlet stream were taken at regular intervals (60 min) and analyzed by NMR spectroscopy. While the highest productivity was obtained at 50 °C with a 0.1 M solution of the substrates and a flow rate of 10 μL min^−1^ (81% conversion; Table 4, entry 1), operating the microreactor **R5** at a lower flow rate (5 μL min^−1^; residence time: 276 min) guaranteed the complete consumption of the reactants (Table 4, entry 2). Under these conditions, the benzoylated benzoin product **3aa** could be isolated in pure form by simple evaporation of the solvent. The long-term stability of **R5** was next examined to establish the effect of the flow regime on the deactivation rate of the PS-BEMP **5**. The analysis of the conversion versus process time plot showed that the steady-state conversion was reached after ca. 3 h at 50 °C and maintained unaltered for about 120 h on stream (Figure 2).

The scope and applicability of the flow cross-benzoin-type reaction were investigated by coupling various α-diketones **1** with aromatic aldehydes **2**. Higher efficiencies were detected with α-diketones **1a–c** displaying electron-neutral and withdrawing groups with expected lower values of reduction potentials (Table 4, entries 3–13), in agreement with the proposed reaction mechanism. The unreactivity of 4,4’-dimethylbenzil (**1d**) seemed to confirm our mechanistic hypothesis (Table 4, entry 14).

**Table 4:** Scope of the continuous-flow benzoin-like reaction.^a^

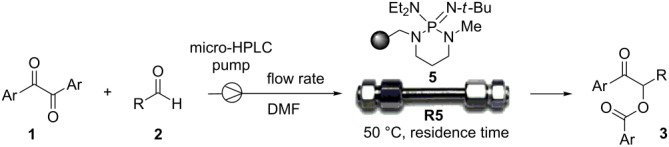						

Entry	Donor (*c* [M])	Acceptor (*c* [M])	Flow rate [μL/min]	Time [min]^b^	Product (Conv. [%])^c^	*P*^d^

1	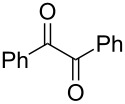 **1a** (0.10)	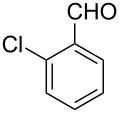 **2a** (0.10)	10	138	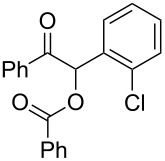 **3aa** (81)	22
2	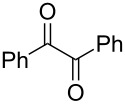 **1a** (0.10)	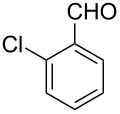 **2a** (0.10)	5	276	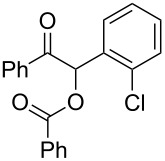 **3aa** (>95)	13
3	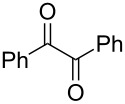 **1a** (0.10)	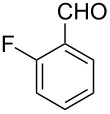 **2b** (0.10)	10	138	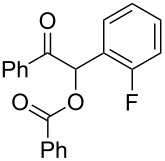 **3ab** (88)	24
4	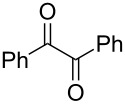 **1a** (0.10)	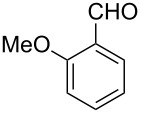 **2c** (0.10)	10	138	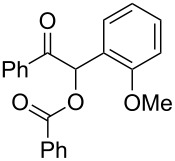 **3ac** (75)	20
5	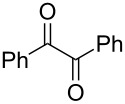 **1a** (0.10)	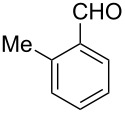 **2d** (0.10)	5	276	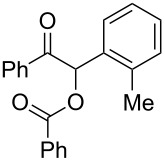 **3ad** (62)	8
6	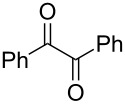 **1a** (0.10)	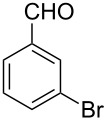 **2e** (0.10)	10	138	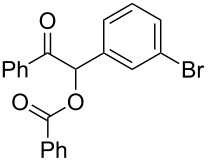 **3ae** (85)	23
7	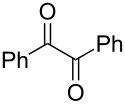 **1a** (0.10)	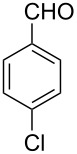 **2f** (0.10)	10	138	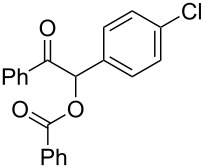 **3af** (90)	25
8	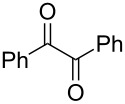 **1a** (0.10)	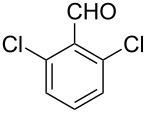 **2g** (0.10)	5	276	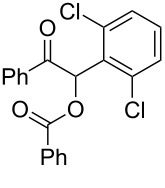 **3ag** (61)	8
9	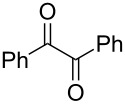 **1a** (0.10)	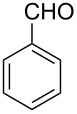 **2h** (0.10)	5	276	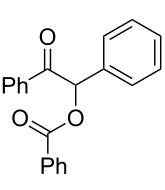 **3ah** (66)	9
10	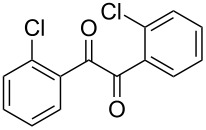 **1b** (0.10)	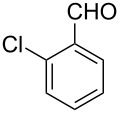 **2a** (0.10)	10	138	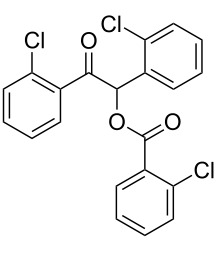 **3ba** (77)	19
11	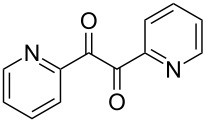 **1c** (0.10)	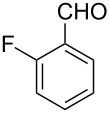 **2b** (0.10)	15	207	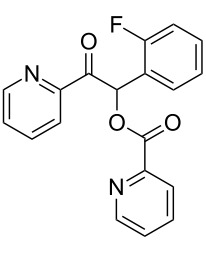 **3cb** (82)	34
12	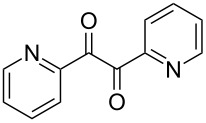 **1c** (0.10)	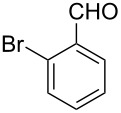 **2i** (0.10)	10	138	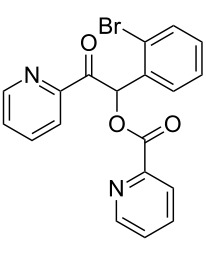 **3ci** (85)	23
13	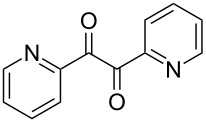 **1c** (0.10)	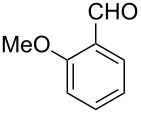 **2c** (0.10)	10	138	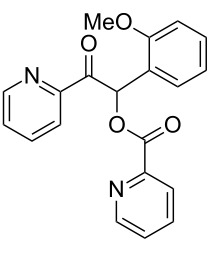 **3cc** (69)	19
14	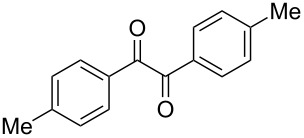 **1d** (0.10)	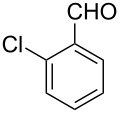 **2a** (0.10)	5	276	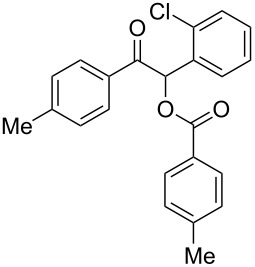 **3da** (<5)	–

^a^See the Experimental section for a description of the experimental setup. Experiments performed for 5 h in steady-state regime. Temperature was measured by a thermometer placed inside the thermostated unit containing the reactor. ^b^Calculated residence time. ^c^Instant conversion in steady-state regime as established by ^1^H NMR analysis. ^d^Productivities are measured in mmol(product) h^−1^ mmol(catalyst)^−1^ × 10^3^.

Following the thread of the previous study on the benzoin condensation, the Stetter-like reaction of benzil (**1a**, 0.1 M) with chalcone **9a** (0.05 M) was optimized at 70 °C with a flow rate of 5 μL min^−1^ (Table 5, entry 1). Because of the partial adsorption of the target 1,4-diketone **10aa** onto the basic packing material **5**, the reactor **R5** was flushed with pure DMF at the end of the coupling experiment, thus permitting the recovery of the whole amount of generated product (see the Experimental section). In general, a lower level of coupling efficiency was detected for the Stetter-like reaction compared to the benzoin condensation as confirmed by the higher residence time (276 min) required to reach satisfactory conversions. Again, benzil **1d** proved to be completely ineffective in the addition to α,β-unsaturated acceptors as well (Table 5, entry 7).

**Table 5:** Scope of the continuous-flow Stetter-like reaction.^a^

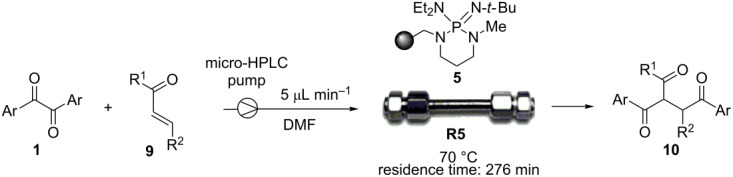				

Entry	Donor (*c* [M])	Acceptor (*c* [M])	Product (conv. [%])^b^	*P*^c^

1	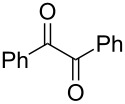 **1a** (0.10)	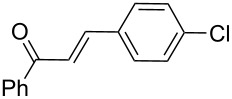 **9a** (0.05)	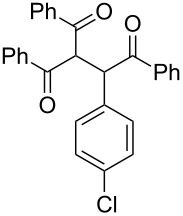 **10aa** (72)	5
2	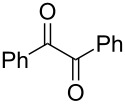 **1a** (0.10)	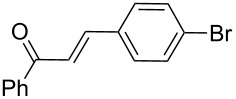 **9b** (0.05)	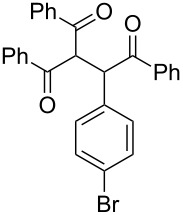 **10ab** (68)	5
3	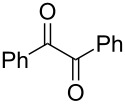 **1a** (0.10)	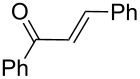 **9c** (0.05)	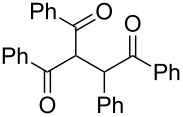 **10ac** (61)	4
4	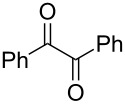 **1a** (0.10)	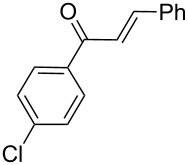 **9d** (0.05)	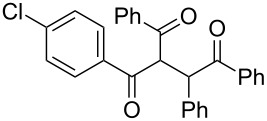 **10ad** (55)^d^	4
5	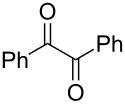 **1a** (0.10)	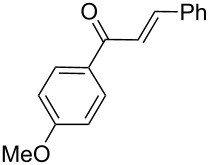 **9e** (0.05)	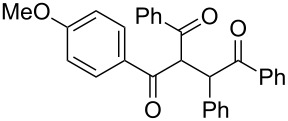 **10ae** (47)^d^	4
6	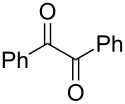 **1a** (0.10)	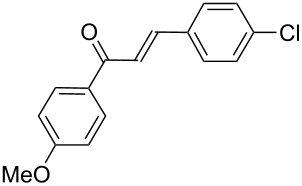 **9f** (0.05)	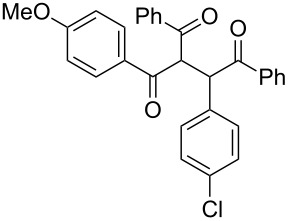 **10af** (42)^d^	3
7	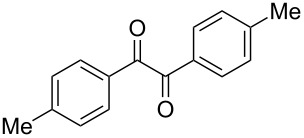 **1d** (0.10)	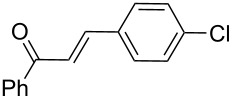 **9a** (0.05)	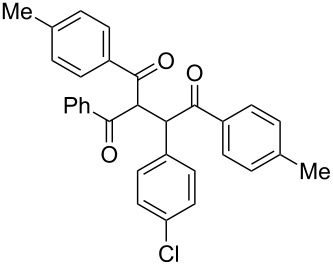 **10da** (<5)	–

^a^See the Experimental section for a description of the experimental setup. Experiments performed for 5 h in steady-state regime. ^b^Instant conversion in steady-state regime as established by ^1^H NMR analysis. ^c^Productivities are measured in mmol(product) h^−1^ mmol(catalyst)^−1^× 10^3^. ^d^Diastereomeric mixture.

## Conclusion

In summary, we have disclosed a practical continuous-flow procedure for the umpolung of aromatic α-diketones and demonstrated its efficacy in the chemoselective synthesis of benzoin- and Stetter-like products (aroylated α-hydroxy ketones and 2-benzoyl-1,4-diones, respectively) through the operation of fixed-bed reactors packed with a readily and commercially available polymer-supported base. Together with the ease of product/promoter separation, an important benefit of the flow regime has been the significant long-term stability of the packing bed (ca. 5 five days on streams). Small-scale reactors have been described in this work; nevertheless, an easy scale-up of the disclosed processes may be envisaged by the numbering up approach.

## Experimental

Liquid aldehydes were freshly distilled before their utilization. Reactions were monitored by TLC on silica gel 60 F_254_ with detection by charring with phosphomolybdic acid. Flash column chromatography was performed on silica gel 60 (230–400 mesh). ^1^H (300 MHz), ^13^C (101 MHz) and ^19^F (376 MHz) NMR spectra were recorded for CDCl_3_ solutions at room temperature unless otherwise specified. Peaks assignments were aided by ^1^H,^1^H COSY and gradient-HMQC experiments. For accurate mass measurements, the compounds were analyzed in positive ion mode by Agilent 6520 HPLC-Chip Q/TOF-MS (nanospray) using a quadrupole, a hexapole, and a time-of-flight unit to produce spectra. The capillary source voltage was set at 1700 V; the gas temperature and drying gas were kept at 350 °C and 5 L/min, respectively. The MS analyzer was externally calibrated with ESI-L low concentration tuning mix from *m*/*z* 118 to 2700 to yield an accuracy below 5 ppm. Accurate mass data were collected by directly infusing samples in 40/60 H_2_O/ACN 0.1% TFA into the system at a flow-rate of 0.4 mL/min. Microwave-assisted reactions were carried out using a single-mode cavity dedicated reactor (Biotage InitiatorTM). Reactions were performed with temperature-controlled programs in glass vials (0.5–2 mL) sealed with a Teflon septum. Temperatures were measured externally by an IR sensor. As described in [32], the system used for continuous-flow reactions was composed of an HPLC pump (Agilent 1100 micro series), an in-line pressure transducer, a thermostated microreactor holder (Peltier unit), a system to collect fractions and a data acquisition system (Agilent ChemStation). The units were connected by peek tubing (internal diameter 0.01 inch from Upchurch Scientific). The system hold-up volume was smaller than 80 µL. The temperature was controlled by inserting a thermometer inside the Peltier unit (temperature measurement error: ±0.5 °C). The supported bases **4**–**8** were purchased from Sigma-Aldrich. All adducts **3** and **10** are known compounds [27–29] apart from compounds **3ab**, **3ag**, **3cb**, **3ci**, and **3cc**.

### Procedure for the model cross-benzoin-like reaction under batch conditions (Table 1)

A mixture of benzil (**1a**, 105 mg, 0.50 mmol), 2-chlorobenzaldehyde (**2a**, 56 μL, 0.50 mmol), the stated base (see Table 1 for molar ratio) and DMF (1.0 mL) was stirred at the stated temperature for the stated time, then filtered and concentrated. The resulting residue was analyzed by ^1^H NMR to determine the conversion. Subsequently, the residue was eluted from a column of silica gel with 20:1 cyclohexane–AcOEt to give isolated **3aa**.

### Procedure for the model Stetter-like reaction under batch conditions (Table 2)

A mixture of benzil (**1a**, 105 mg, 0.50 mmol), (*E*)-3-(4-chlorophenyl)-1-phenylprop-2-en-1-one (**9a**, 121 mg, 0.50 mmol), PS-BEMP **5** (see Table 2 for molar ratio) and DMF (1.0 mL) was stirred at the stated temperature for the stated time, then filtered and concentrated. The resulting residue was analyzed by ^1^H NMR to determine the conversion. Subsequently, the residue was eluted from a column of silica gel with 13:1 cyclohexane–AcOEt to give isolated **10aa**.

### Trapping experiment (Scheme 2)

A mixture of benzil (**1a**, 210 mg, 1.00 mmol), PS-BEMP **5** (454 mg, 1.00 mmol) and DMF (2 mL) was stirred at 50 °C for 30 min then acetic anhydride (0.94 mL, 10.0 mmol) was added in one portion. The reaction mixture was stirred at 50 °C for 2 h, then cooled to room temperature, filtered, concentrated, and eluted from a column of silica gel with 7:1 cyclohexane–AcOEt to give (*Z*)-1,2-diphenylethene-1,2-diyl diacetate **11** as a white amorphous solid (17 mg, 6%). ^1^H NMR (300 MHz, CDCl_3_) δ 7.30–7.16 (m, 10H, Ar), 2.21 (s, 6H, CH_3_); ^13^C{^1^H} NMR (101 MHz, CDCl_3_) δ 168.1, 138.5, 133.0, 128.8, 128.8, 128.2, 20.7; HRMS–ESI/Q-TOF (*m*/*z*): [M]^+^ calcd for C_18_H_16_O_4_: 296.1049; found: 296.1105.

### Determination of microreactor void-volume

Microreactor void volume (*V*_0_) was determined by pycnometry [31]. This method consists in filling the microreactor successively with two distinct solvents (solvent 1: water; solvent 2: *n*-hexane) and weighing the filled microreactors accurately. Simple math shows that [33]: V_0_ = (ω_1_ − ω_2_) / (δ_1_ − δ_2_), where ω_1_ and ω_2_ are the weights of the microreactor filled with solvents 1 and 2 and δ_1_ and δ_2_ the densities of the solvents.

### Continuous-flow cross-benzoin-like reactions (Table 4)

Microreactor **R5** was fed with a DMF solution of α-diketone **1** and aldehyde **2** (see Table 4 for molarity concentrations), and operated at the stated temperature and the stated flow rate for 5 h under steady-state conditions. Instant conversion was determined (^1^H NMR analysis) every hour by taking a sample of the eluate. The collected solution was finally concentrated and eluted from a column of silica gel with the suitable elution system to give the corresponding aroylated α-hydroxy ketone **3**.

The long-term stability experiment was performed using benzil (**1a**, 0.10 M) and 2-chlorobenzaldehyde (**2a**, 0. 10 M) as the substrates; microreactor **R5** was operated at 50 °C with a flow rate of 5 μL min^−1^ for 150 h. After the achievement of the steady-state regime (ca. 3 h), an almost full conversion of **1a** (>95%) was maintained for ca. 120 h, while a progressive loss of catalytic activity was observed after that time.

### Continuous-flow Stetter-like reactions (Table 5)

Microreactor **R5** was fed with a DMF solution of α-diketone **1** (0.10 M) and chalcone **9** (0.05 M), and operated at 70 °C with a flow rate of 5 μL min^−1^ for 5 h under steady-state conditions. After that time, the reactor was flushed at room temperature with pure DMF for an additional 5 h. The collected solution was finally concentrated and eluted from a column of silica gel with the suitable elution system to give the corresponding 2-benzoyl-1,4-dione **10**.

**1-(2-Fluorophenyl)-2-oxo-2-phenylethyl benzoate (3ab). **^1^H NMR (300 MHz, CDCl_3_) δ 8.15–8.06 (m, 2H, Ar), 8.06–7.97 (m, 2H, Ar), 7.61–7.50 (m, 3 H, Ar), 7.50–7.40 (m, 5H, Ar, H-1), 7.40–7.31 (m, 1H, Ar), 7.21–7.05 (m, 2H, Ar); ^13^C{^1^H} NMR (101 MHz, CDCl_3_) δ 192.9, 165.9, 160.2 (d, *J* = 250 Hz), 134.4, 133.9, 133.5, 131.5 (d, *J* = 8.4 Hz), 130.1 (d, *J* = 2.5 Hz), 129.3, 129.0, 128.9, 128.7, 128.5, 125.0 (d, *J* = 3.3 Hz), 121.4 (d, *J* = 14 Hz), 116.3 (d, *J* = 22 Hz), 70.7; ^19^F NMR (376 MHz, CDCl_3_) δ −116.7 to −116.8 (m); HRMS–ESI/Q-TOF (*m*/*z*): [M + Na]^+^ calcd for C_21_H_15_FNaO_3_: 357.0903; found: 357.0988.

**1-(2,6-Dichlorophenyl)-2-oxo-2-phenylethyl benzoate (3ag). **^1^H NMR (300 MHz, CDCl_3_) δ 8.19–8.11 (m, 2H, Ar), 7.85–7.78 (m, 3H, Ar, H-1), 7.62–7.51 (m, 1H, Ar), 7.51–7.41 (m, 3H, Ar), 7.41–7.31 (m, 4H, Ar), 7.27–7.18 (m, 1H, Ar); ^13^C{^1^H} NMR (101 MHz, CDCl_3_) δ 192.8, 165.2, 136.7, 134.9, 133.4, 133.2, 132.0, 131.0, 130.2, 129.3, 129.3, 128.5, 128.4, 128.08, 75.1; HRMS–ESI/Q-TOF (*m*/*z*): [M + Na]^+^ calcd for C_21_H_14_Cl_2_NaO_3_: 407.0218; found: 407.0301.

**1-(2-Fluorophenyl)-2-oxo-2-(pyridin-2-yl)ethyl picolinate (3cb). **^1^H NMR (300 MHz, CDCl_3_) δ 8.81–8.72 (m, 1H, Ar), 8.59 (m, 1H, Ar), 8.21–8.13 (m, 1H, Ar), 8.08–8.00 (m, 1H, Ar), 7.97 (s, 1H, H-1), 7.86–7.73 (m, 2H, Ar), 7.57–7.36 (m, 3H, Ar), 7.36–7.26 (m, 1H, Ar), 7.16–7.01 (m, 2H, Ar); ^13^C{^1^H} NMR (101 MHz, CDCl_3_) δ 193.3, 164.4, 161.0 (d, *J* = 250 Hz), 159.7, 151.2, 150.1, 149.1, 147.6, 137.0, 136.9, 131.2 (d, *J* = 8.4 Hz), 130.7 (d, *J* = 2.3 Hz), 127.7, 127.1, 125.70, 124.4 (d, *J* = 3.7 Hz), 122.9, 121.4 (d, *J* = 14 Hz), 116.2 (d, *J* = 22 Hz), 72.4; ^19^F NMR (376 MHz, CDCl_3_) δ −115.2 to −115.3 (m); HRMS–ESI/Q-TOF (*m*/*z*): [M + H]^+^ calcd for C_19_H_14_FN_2_O_3_: 337.0988; found: 337.0908.

**1-(2-Bromophenyl)-2-oxo-2-(pyridin-2-yl)ethyl picolinate (3ci). **^1^H NMR (300 MHz, CDCl_3_) δ 8.81–8.73 (m, 1H, Ar), 8.63–8.55 (m, 1H, Ar), 8.20–8.13 (m, 1H, Ar), 8.09–8.02 (m, 2H, Ar, H-1), 7.86–7.74 (m, 2H, Ar), 7.64 (m, 1H, Ar), 7.50–7.36 (m, 3H, Ar), 7.28–7.13 (m, 2H, Ar); ^13^C{^1^H} NMR (101 MHz, CDCl_3_) δ 194.0, 164.3, 151.3, 150.2, 149.2, 147.6, 137.0, 136.9, 133.8, 133.7, 130.6, 130.5, 127.8, 127.7, 127.1, 125.8, 122.8, 78.0; HRMS–ESI/Q-TOF (*m*/*z*): [M + H]^+^ calcd for C_19_H_14_BrN_2_O_3_: 397.0188; found: 397.0225.

**1-(2-Methoxyphenyl)-2-oxo-2-(pyridin-2-yl)ethyl picolinate (3cc). **^1^H NMR (300 MHz, CDCl_3_) δ 8.79–8.68 (m, 1H, Ar), 8.59–8.46 (m, 1H, Ar), 8.17–8.07 (m, 1H, Ar), 8.06–7.99 (m, 2H, Ar, H-1), 7.82–7.70 (m, 2H, Ar), 7.46–7.39 (m, 1H, Ar), 7.39–7.29 (m, 2H, Ar), 7.29–7.23 (m, 1H, Ar), 6.93–6.83 (m, 2H, Ar), 3.83 (s, 3H, CH_3_); ^13^C{^1^H} NMR (101 MHz, CDCl_3_) δ 194.7, 164.6, 157.8, 151.9, 150.1, 149.0, 147.9, 137.0, 136.8, 130.7, 130.2, 127.4, 127.0, 125.6, 122.7, 122.6, 120.8, 111.7, 73.4, 55.9; HRMS–ESI/Q-TOF (*m*/*z*): [M + H]^+^ calcd for C_20_H_17_N_2_O_4_: 349.1188; found: 349.1105.

## Supporting Information

File 1 NMR spectra of new compounds.
