# Supplementary material for: Electron-transfer-initiated benzoin- and Stetter-like reactions in packed-bed reactors for process intensification
Source: Beilstein J Org Chem. 2016 Dec 13;12:2719–30. doi: 10.3762/bjoc.12.268 (PMC5238549; doi:10.3762/bjoc.12.268)

Supporting Information

for

Electron-transfer-initiated benzoin- and Stetter-like reactions in packed-bed reactors for process intensification

Anna Zaghi, Daniele Ragno, Graziano Di Carmine, Carmela De Risi, Olga Bortolini, Pier Paolo Giovannini, Giancarlo Fantin, and Alessandro Massi*

Address: Dipartimento di Scienze Chimiche e Farmaceutiche, Università di Ferrara, Via Fossato di Mortara 17, I-44121 Ferrara (Italy)

Email: Alessandro Massi - alessandro.massi@unife.it

*Corresponding author

NMR spectra of new compounds

^{1}H (300 MHz), ^{13}C (101 MHz), and ^{19}F (376 MHz) spectra ($CDCl_3$) of 3ab.

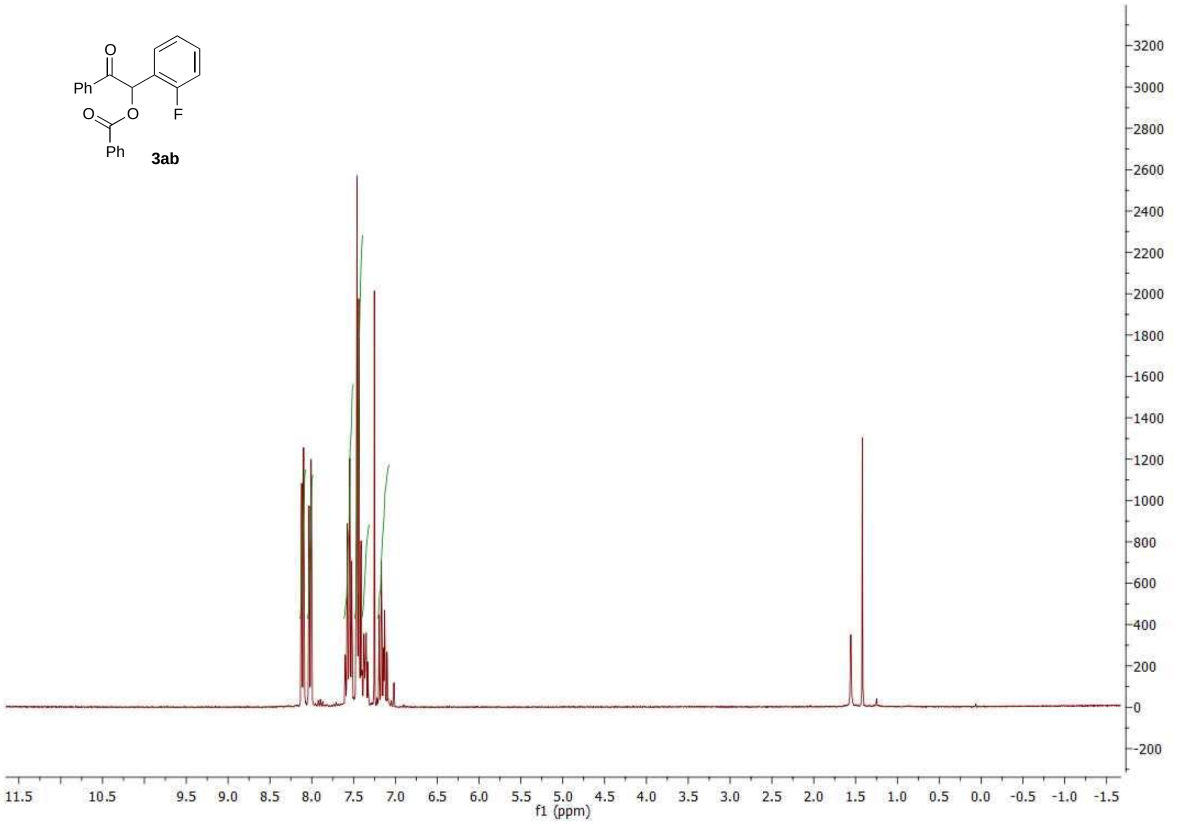

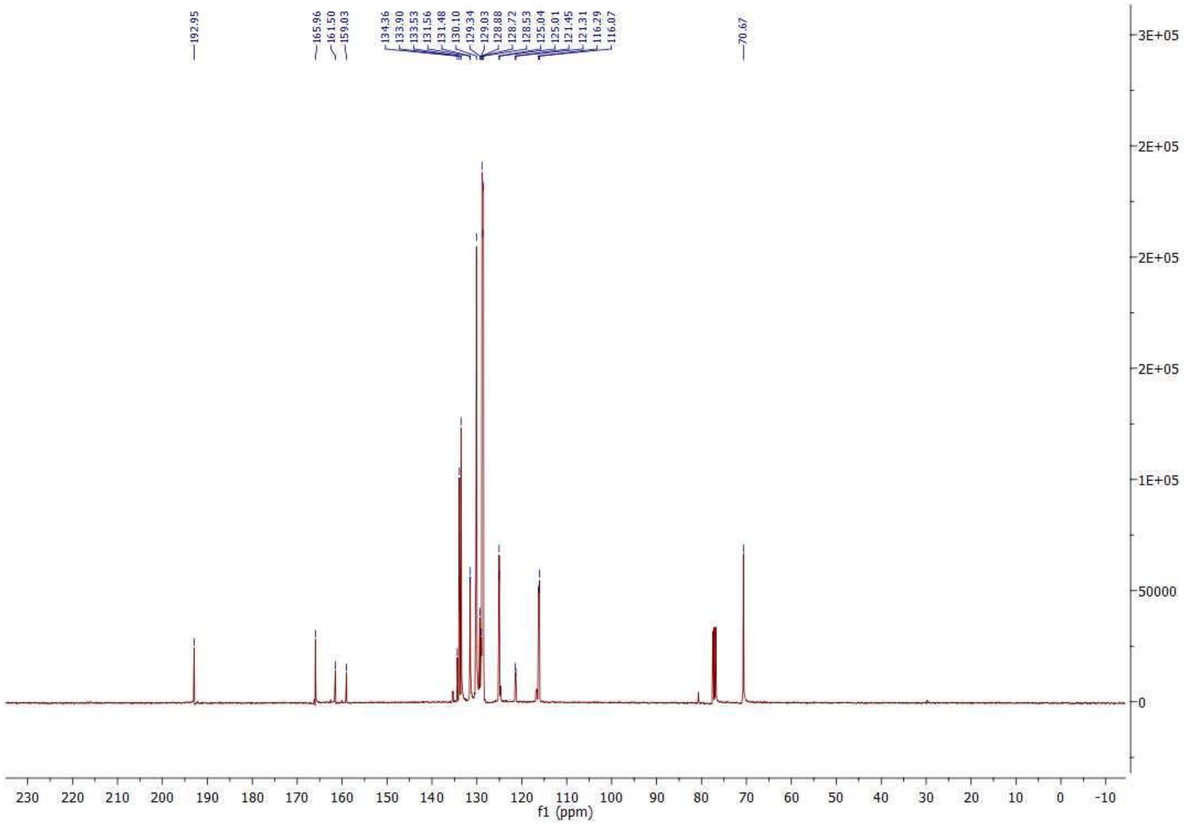

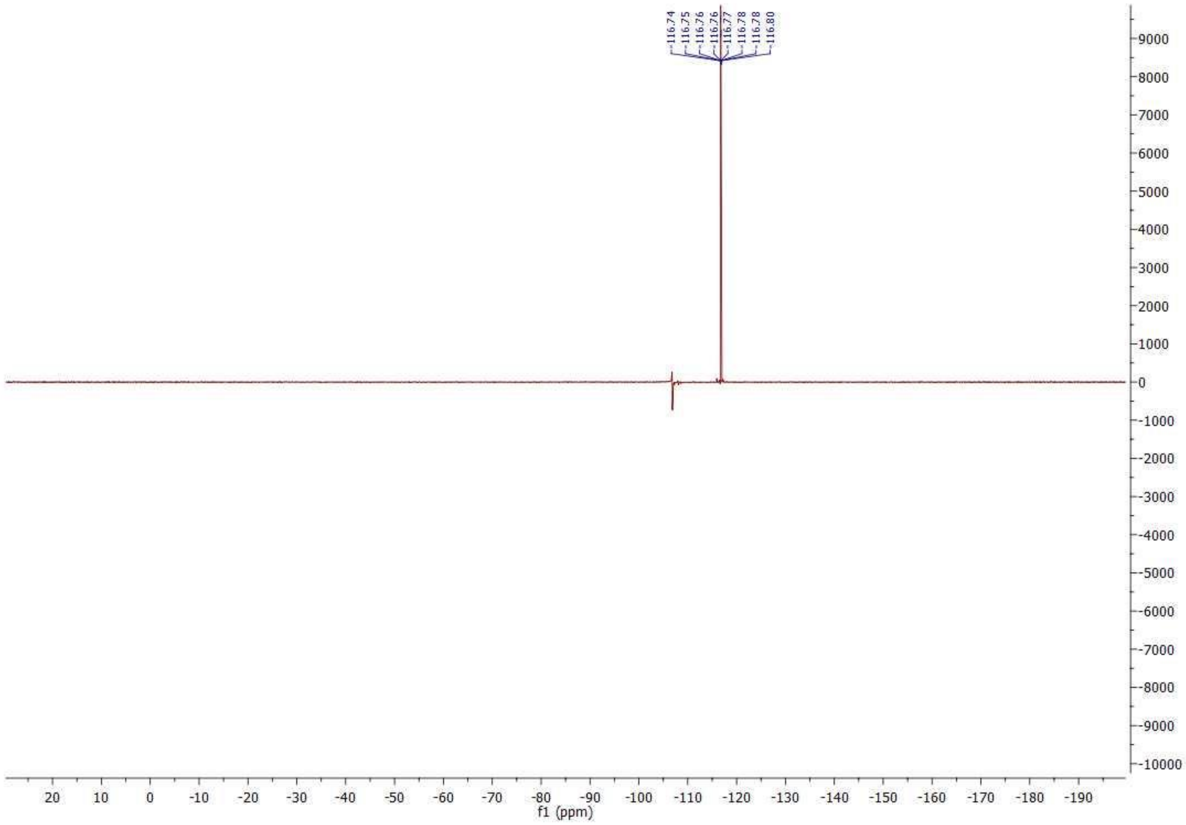
-116.74
-116.75
-116.76
-116.76
-116.77
-116.78
-116.78
-116.80
f1 (ppm)

^{1}H (300 MHz) and ^{13}C (101 MHz) spectra ($CDCl_3$) of 3ag.

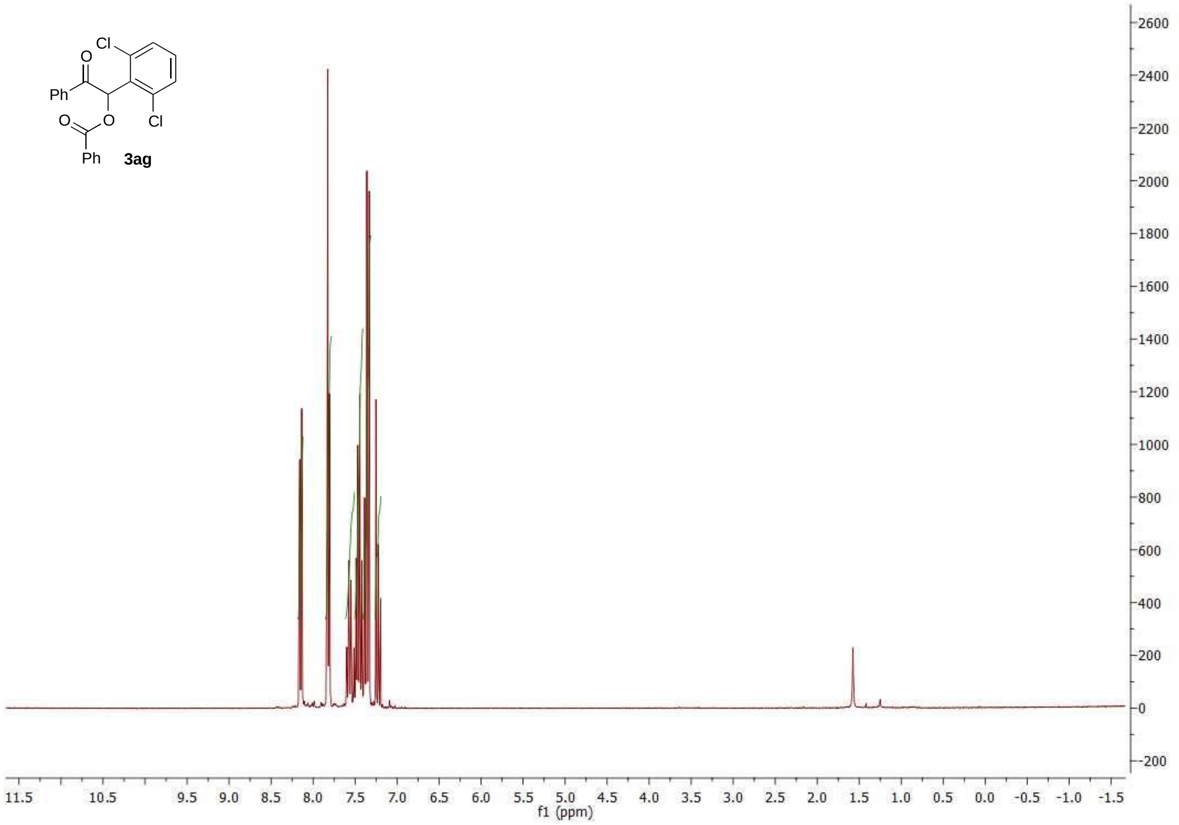

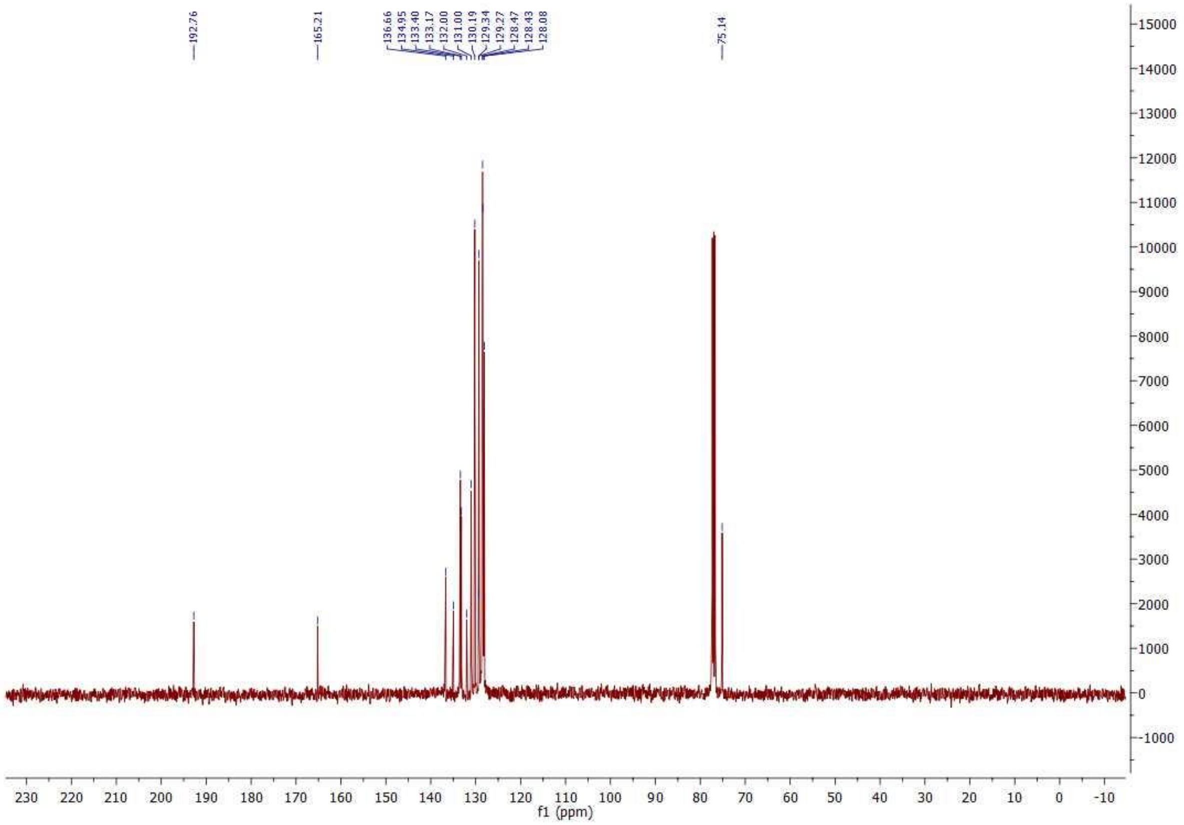

^{1}H (300 MHz), ^{13}C (101 MHz), and ^{19}F (376 MHz) spectra ($CDCl_3$) of 3cb.

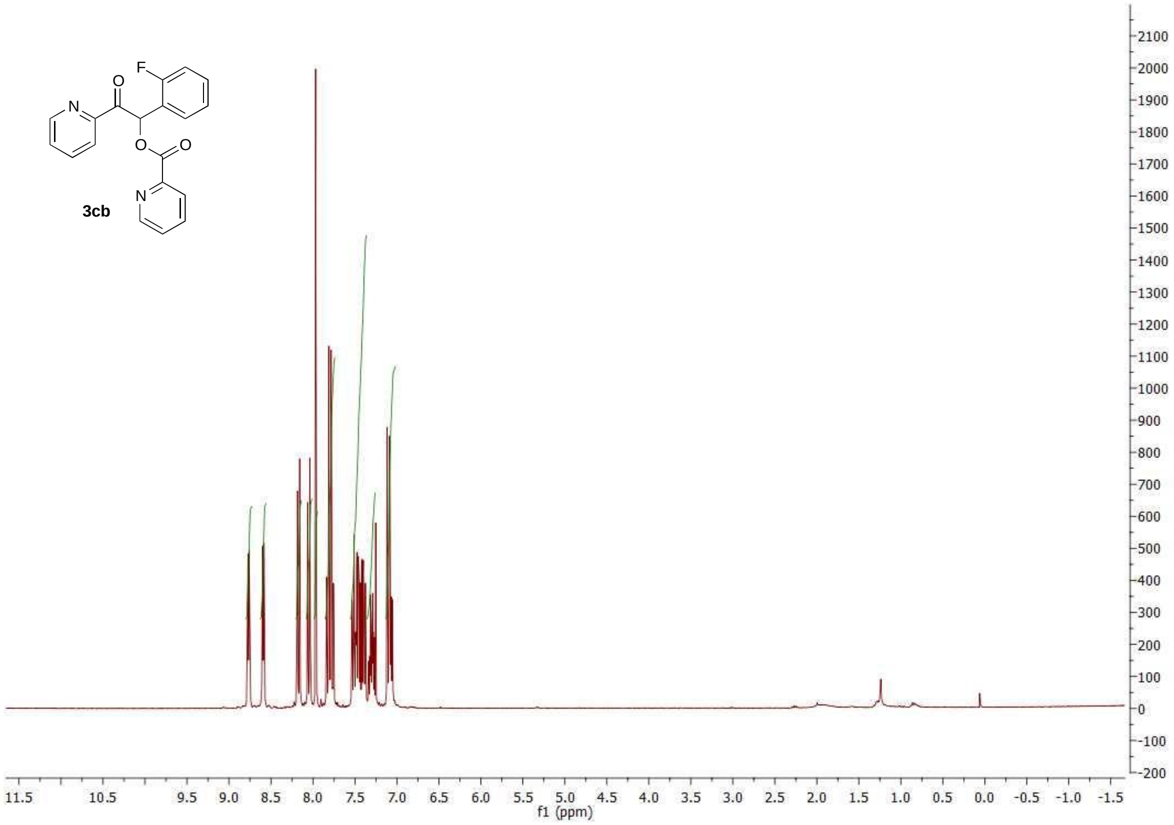

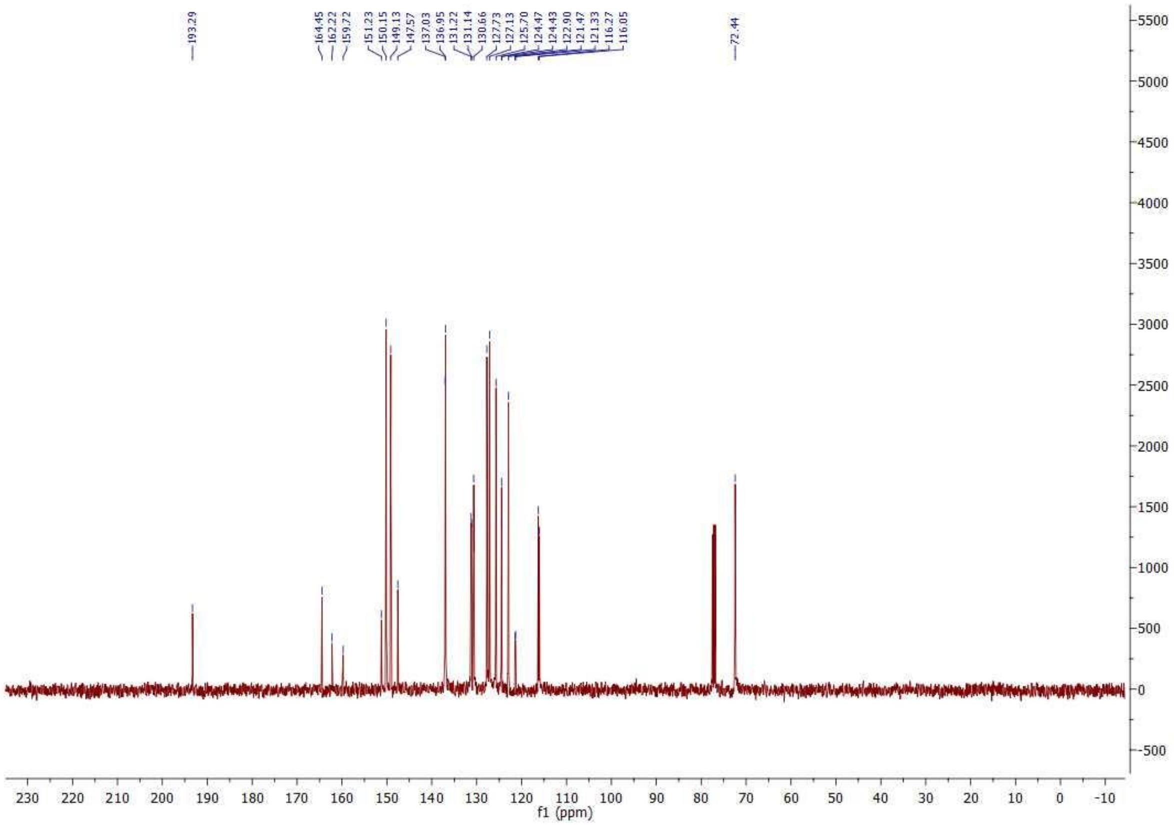

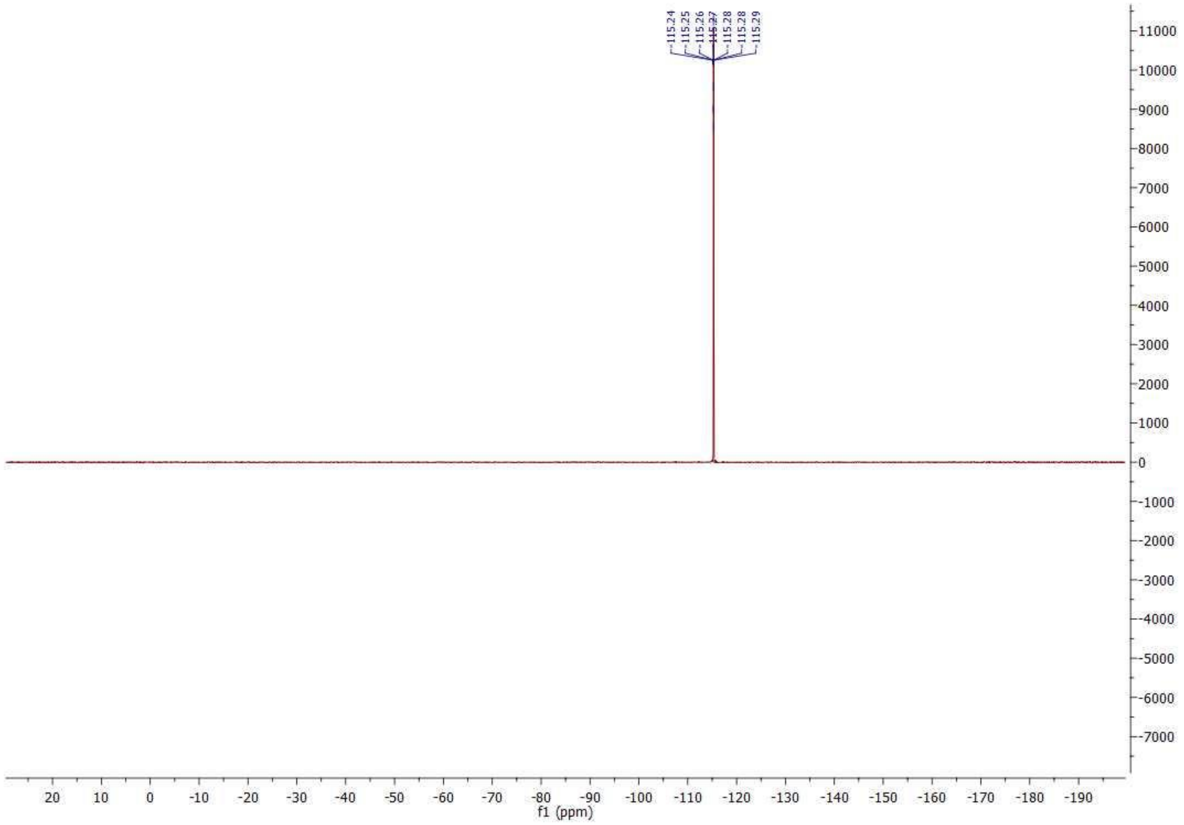
-115.24
-115.25
-115.26
-115.27
-115.28
-115.28
-115.29
f1 (ppm)

^{1}H (300 MHz) and ^{13}C (101 MHz) spectra ($CDCl_3$) of 3ci.

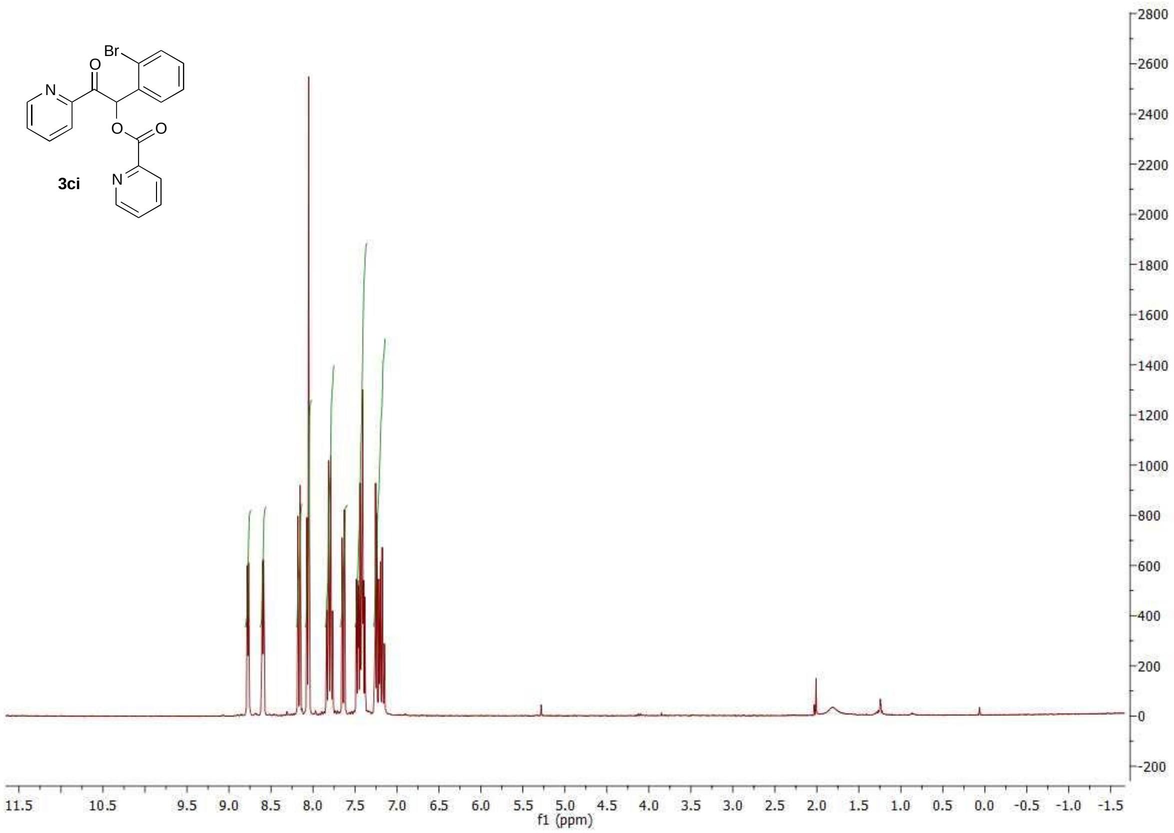

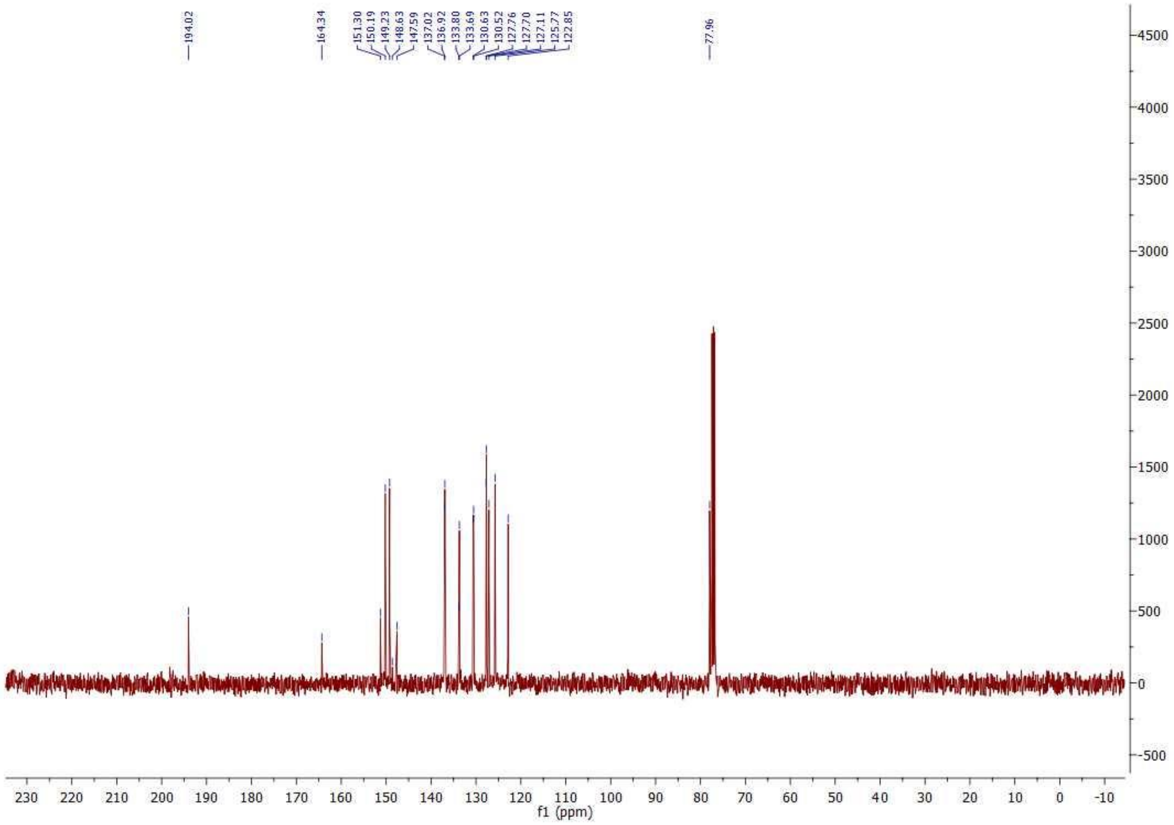

^{1}H (300 MHz) and ^{13}C (101 MHz) spectra ($CDCl_3$) of 3cc.

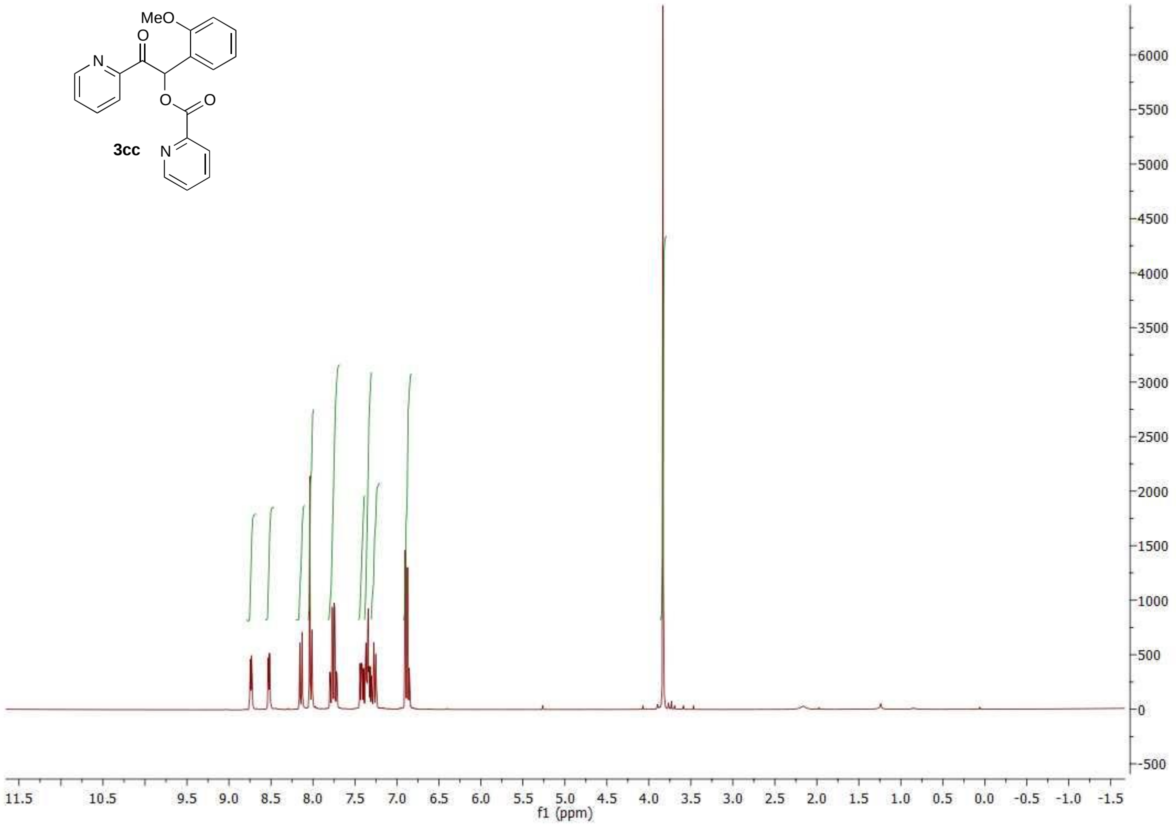

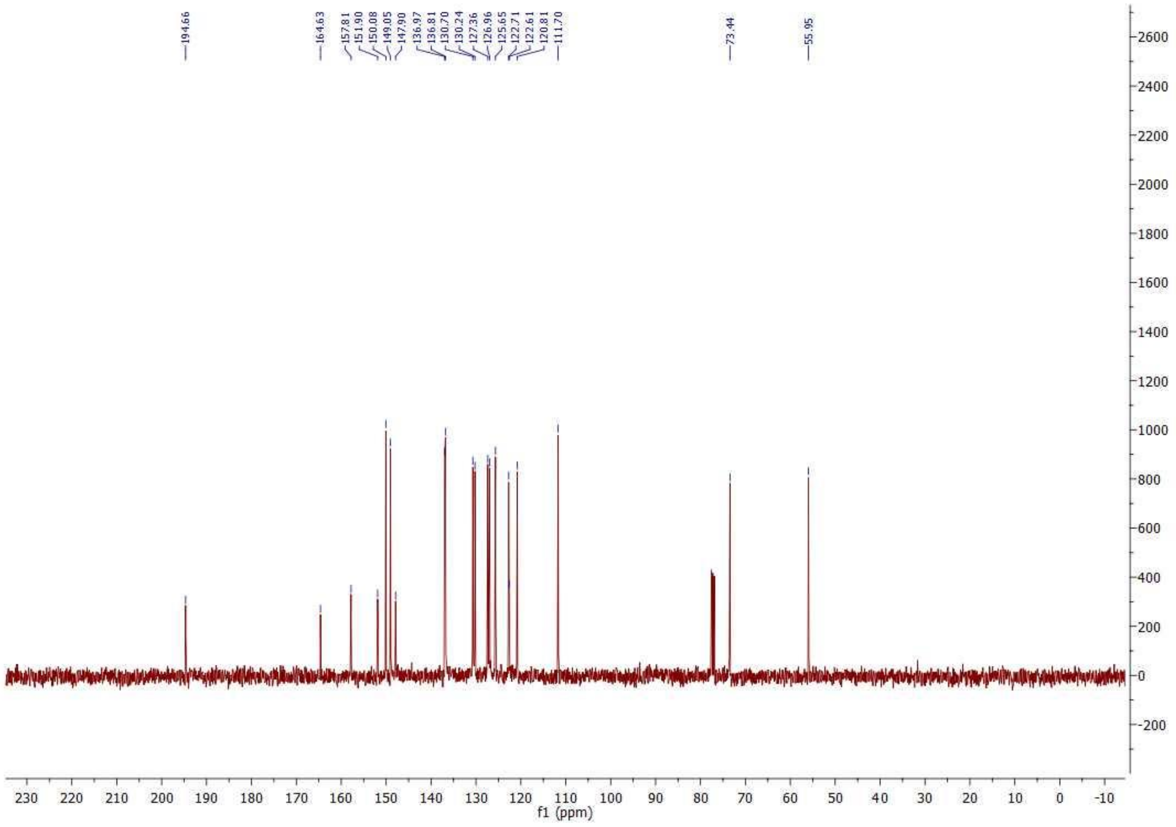

Supplement: File 1 — NMR spectra of new compounds. [file Beilstein_J_Org_Chem-12-2719-s001.pdf]
